# Different Gestational Diabetes Phenotypes: Which Insulin Regimen Fits Better?

**DOI:** 10.3389/fendo.2021.630903

**Published:** 2021-03-09

**Authors:** Federico Mecacci, Federica Lisi, Silvia Vannuccini, Serena Ottanelli, Marianna Pina Rambaldi, Caterina Serena, Serena Simeone, Felice Petraglia

**Affiliations:** ^1^ Department of Biomedical, Experimental and Clinical Sciences, University of Florence, Careggi University Hospital, Florence, Italy; ^2^ High Risk Pregnancy Unit, Careggi University Hospital, Florence, Italy; ^3^ Department of Molecular and Developmental Medicine, University of Siena, Siena, Italy

**Keywords:** body mass index (BMI), diet, gestational diabetes mellitus (GDM), glycemic control, hyperglycemia, insulin analogues, obesity, oral glucose tolerance test (OGTT)

## Abstract

**Objective:**

Maternal characteristics and OGTT values of pregnancies complicated by gestational diabetes mellitus (GDM) were evaluated according to treatment strategies. The goal was to identify different maternal phenotypes in order to predict the appropriate treatment strategy.

**Methods:**

We conducted a retrospective study among 1,974 pregnant women followed up for GDM in a tertiary referral hospital for high-risk pregnancies (Careggi University Hospital, Florence, Italy) from 2013 to 2018. We compared nutritional therapy (NT) alone (n = 962) *versus* NT and insulin analogues (n = 1,012) group. Then, we focused on different insulin analogues groups: long acting (D), rapid acting (R), both D and R. We compared maternal characteristics of the three groups, detecting which factors may predict the use of rapid or long-acting insulin analogue alone *versus* combined therapy.

**Results:**

Among women included in the analysis, 51.3% of them needed insulin therapy for glycemic control: 61.8% D, 28.3% combined D and R, and 9.9% R alone. Age >35 years, pre-pregnancy BMI >30, family history of diabetes, previous GDM, altered fasting plasma glucose (FPG), hypothyroidism, and assisted reproductive technologies (ART) were identified as maternal variables significantly associated with the need of insulin therapy. Altered 1-h and 2-h glucose plasma glucose level at OGTT, age >35 years, and previous GDM were found as independent predicting factors for the use of combined therapy with rapid and long acting analogues for glycemic control. On the contrary, pre-pregnancy BMI <25 and normal fasting plasma glucose values at OGTT were found to be significantly associated to the use of rapid insulin analogue only.

**Conclusion:**

A number of maternal and metabolic variables may be identified at the diagnosis of GDM, in order to identify different GDM phenotypes requiring a personalized treatment for glycemic control.

## Introduction

Gestational diabetes mellitus (GDM) is considered a major global health problem because of its increasing prevalence and the well-known association between hyperglycemia in pregnancy and feto-maternal morbidity (1, 2). Fetal outcomes correlate with maternal hyperglycemia severity, determining not only perinatal and neonatal complications in the short term (such as macrosomia, shoulder dystocia, respiratory distress, hypoglycemia, polycythemia, and hyperbilirubinemia), but also long-term sequelae with increased risk of obesity and diabetes later in life (3). Therefore, it is mandatory to perform a timely diagnosis of GDM and to improve therapeutic strategies in order to achieve the optimal glycemic control during pregnancy in the least amount of time.

Insulin therapy is one of the key strategies in managing diabetes in pregnancy, when nutritional therapy (NT) and lifestyle changes alone are not enough for an adequate glycemic control. In the last 10 years short and long-acting insulin analogues have replaced human-derived insulin, with a number of advantages for glycemic control. Short-acting analogues (Lispro and Aspart) have faster onset and shorter duration of action determining a more overlapping peak to the one of the endogenous insulin, reducing the risk of post-meal hypoglycemia and improving glycemic control in women with GDM (4, 5). The long-acting analogues (Glargine and Detemir) and their good pharmacokinetic characteristics allow to control basal glycemia providing a flat and protracted pharmacodynamic profile (4, 6). Their use has increased in the last decade because of the rising attention to fasting glucose profile control rather than post meal glycemic values. Maternal 1-h postprandial glycemic peak is one of the best predictor of fetal macrosomia, but fasting glucose levels also significantly influence perinatal and neonatal outcome (7, 8).

Choosing the best therapeutic strategy in terms of type and timing of insulin therapy is essential to improve glycemic control in pregnancy. A number of maternal features and clinical data at diagnosis of GDM are reported to be associated with failure of NT and subsequent need for pharmacological treatment. For instance, early diagnosis of GDM (<24 weeks of gestation), older maternal age, pre-pregnancy BMI>30, previous GDM, family history of diabetes are reported as associated factors to insulin treatment (9–12). Biochemical parameters indicating poor metabolic glycemic control at the diagnosis of GDM can also predict the need of insulin therapy: HbA1c at diagnosis >5.5% and elevated 1-h and 2-h plasma glucose level at OGTT has been reported to be predictors of pharmacological treatment (13, 14).

The aim of the present study is to analyze maternal characteristics and OGTT values in pregnancies complicated by GDM according to different treatment strategies (diet alone or diet plus various combination of long- and short-term insulin analogues). The goal is to identify different maternal phenotypes in order to plan, since the diagnosis of GDM, the appropriate treatment strategy.

## Materials and Methods

We performed a retrospective cohort study among 1,974 women with singleton pregnancy affected by GDM, who were referred to High Risk Pregnancy Unit in Careggi University Hospital (Florence, Italy) from 2013 to 2018. We compared women treated with nutritional therapy (NT) *versus* women treated with NT and insulin analogues. Women with pre-pregnancy diabetes or glucose intolerance, previous insulin treatment, current consuming oral glucose-lowering drugs, such as metformin, and twin pregnancies were excluded from the study.

According to Figo guidelines (3) all women underwent universal screening for GDM by 75 g oral glucose tolerance test (OGTT) at 24–28 weeks of gestation. Women considered at high risk to develop GDM [previous GDM, BMI >30, fasting plasma glucose (FPG) 100–125 mg/dl at first trimester blood test] were screened at 16–18 weeks and in case of normal glucose values, they repeated OGTT at 24–28 weeks (15). The diagnosis of GDM was made if one or more of the glucose values was equal or over the cutoff proposed by IADPSG Consensus Panel 2010 (16).

After diagnosis of GDM, patients were educated to self-monitoring of blood glucose. Finger sticks were obtained four to seven times a day: preprandial, postprandial (1 h and 2 h after breakfast, lunch, or dinner) and during the night. A nutritional counseling with an expert dietician devoted to GDM patients was provided every 2 weeks. Nutrition plan included a diet with adequate energy content, macronutrient distribution, quality and amount, in order to preserve mother’s weight gain and fetal growth, as well as maintaining near-normoglycemia and avoiding the development of ketone bodies. Also lifestyle interventions were proposed at every consultation.

The glucose reference values recommended by Italian National Guidelines were used: fasting (90 mg/dl), 1 h (130 mg/dl), and 2 h after a meal (120 mg/dl) (15). Insulin treatment was started with long-acting analogue (*Detemir*) (D) when glycemic values at fasting or during night were > 90 mg/dl in more than half of measurements during 2 weeks assessment. We prescribed Detemir in the morning or in the evening considering if glucose values were more altered during day or nighttime, but we often prescribed two shot-therapy with injections every 12 h. We used rapid analogue (*Aspart, Lispro*) (R) only when we were unable to normalize postprandial blood glucose levels with Detemir or when we had optimal fasting glycemic control but high postprandial values, despite an adequate NT: in this latter case we used only rapid insulin analogue.

Clinical data were extracted by reviewing obstetric records of all women followed up in our GDM clinic in a standardized electronic database. Collected data included maternal characteristics (age, ethnicity, BMI, family history, obstetric history, mode of conception, pre-pregnancy diseases), OGTT values, and GDM management (diet *vs* insulin treatment; short- and long-acting insulin analogues) until delivery. The last reported treatment was considered to classify each case according to the type of therapeutic approach for glycemic control. We also collected data on delivery and neonatal outcome, including gestational age at delivery, induction of labor, mode of delivery, small for gestational age (SGA), and large for gestational age (LGA) neonates, birthweight >4,000g, Apgar score at 10 min. In our department, according to national guidelines, if glycemic values are compensated by only NT, induction of labor is scheduled for GDM after 40 weeks + 6 days, whereas in case of insulin treatment women are induced by 40 weeks or earlier if glycemic values are not within the ranges.

The research protocol involved only existing records, based on information routinely collected and stored in a de-identified dataset and hence was considered exempt from ethical review board approval. However, all women referred to our GDM clinic signed an informed consent for clinical data treatment.

We analyzed maternal characteristics between the group of women treated with NT alone *vs* those treated with NT and insulin, identifying those maternal factors and metabolic variables at OGTT which could predict the need and type of insulin therapy. Then, among women treated with NT and insulin, we identified three groups under treatment with different types of insulin analogues: long-acting analogue, long- and rapid-acting analogues, only rapid-acting analogue. We compared maternal characteristics of the three groups, detecting which factors may predict the use of rapid or long-acting insulin analogue alone *versus* combined therapy using. Also the perinatal outcomes were compared between the group of women under NT *vs* those under NT and insulin treatment, and differentiating among the three insulin analogues treatment groups.

The collected data were entered in an electronic database and analyzed using the software SPSS (Statistical Package for Social Science) (IBM SPSS Statistics 23, IBM Corporation). Percentages, means, and standard deviations (SD) have been extrapolated, followed by the creation of charts and tables for a descriptive analysis. Data distribution was evaluated by the D’Agostino & Pearson omnibus normality test. Baseline characteristics were compared by using chi-square test with continuity correction or Fisher’s exact test for binomial variables. Mann-Whitney U, t test for unpaired or one-way ANOVA were used for continuous variables. A multivariable stepwise (conditional) logistic regression was used to identify the variables associated with NT *vs* insulin therapy and, similarly, those related to the different regimen of insulin therapy. Odds Ratios (OR) and 95% CI were calculated. A receiving operating curve (ROC) analysis was further performed on combined maternal and metabolic variables, significantly associated to the need of insulin treatment. A p value <0.05 was considered statistically significant.

## Results

In the study group 962 (48.7%) women were treated with NT alone, whereas 1,012 (51.3%) women received diet and insulin therapy. Maternal characteristics of the two groups are reported in **Table 1**. Regarding timing of GDM diagnosis, 11.2% (n = 221) of women had a positive OGTT at 16–18 weeks. Comparing the diet and insulin groups, we observed that patients who needed insulin therapy had higher pre-pregnancy BMI (23.9 ± 4.91 *vs* 25.2 ± 5.49, p < 0.001) and were more likely to be obese (6.3 *vs* 12%, p < 0.001). They had also more frequently family history of diabetes (23.2 *vs* 30.5%, p < 0.001) and previous GDM (8.8 *vs* 14.9%, p < 0.001). Furthermore, patients in therapy with insulin were significantly more affected by chronic hypertension (0.5 *vs* 3.2%, p < 0.001) and hypothyroidism (6.1 *vs* 9.9%, p 0.002), and their pregnancy was more likely achieved using assisted reproductive technology (ART) (4.2 *vs* 7.3%, p 0.003). We didn’t find any difference in age, ethnicity, and rate of previous macrosomia between the two groups.

**Table 1:** Maternal characteristics, including anthropometric data, family and clinical history, of women with GDM treated with only diet and those requiring also insulin.

Maternal characteristics	Dietn = 962	Diet + Insulin n = 1,012	p value
Age (y)	33.9 ± 5.33	34.6 ± 5,4	ns
Caucasian ethnicity	815 (84.8%)	850 (84%)	ns
Pre-pregnancy BMI	23.9 ± 4.91	25.2 ± 5.49	<0.001
BMI >30	61 (6.3%)	121 (12.0%)	<0.001
Family history DM	223 (23.2%)	309 (30.5%)	<0.001
Pluriparity	372 (40%)	434 (45.3%)	0.02
ART pregnancy	40 (4.2%)	74 (7.3%)	0.003
Previous GDM	85 (8.8%)	151 (14.9%)	<0.001
Previous macrosomia	11 (1.1%)	14 (1.4%)	ns
Hypothyroidism	56 (6.1%)	100 (9.9%)	0.002
Chronic Hypertension	5 (0.5%)	32 (3.2%)	<0.001

Values are expressed as mean ± SD or n(%). BMI, body mass index; DM, diabetes mellitus; ART, assisted reproductive technologies; GDM, gestational diabetes mellitus.

When we compared OGTT values between the two groups, we found that the insulin therapy group had higher fasting plasma glucose (FPG) values (86.7 ± 23.4 mg/dl *vs* 90.1 ± 9.7 mg/dl, p < 0.001), higher 1-h glucose values (171.5 ± 21.1 mg/dl *vs* 177.7 ± 21.2 mg/dl, p < 0,001), and higher numbers of altered glucose values at OGTT (n.3 altered values 2.6 *vs* 8.3%, p < 0.001) (**Table 2**).

**Table 2:** OGTT values among women with GDM on diet *vs* diet + insulin treatment.

	Dietn = 962	Diet + Insulinn = 1,012	p value
**OGTT values**			
0’	86.71 ± 23.39	90.07 ± 9.70	<0.001
60’	171.48 ± 21.12	177.71 ± 21.16	<0.001
120’	138.06 ± 30.08	140.45 ± 33.29	ns
**Which altered value at OGTT**			
1st	342 (35.6%)	492 (48.6%)	<0.001
2nd	494 (51.4%)	564 (55.7%)	ns
3rd	362 (37.6%)	381 (37.5%)	ns
**How many altered values at OGTT**			
n. 1	737 (76.6%)	641 (63.3%)	<0.001
n. 2	193 (20.1%)	272 (26.9%)	<0.001
n. 3	25 (2.6%)	84 (8.3%)	<0.001

Values are expressed as mean ± SD or n(%). OGTT, oral glucose tolerance test.

**Figure 1:**
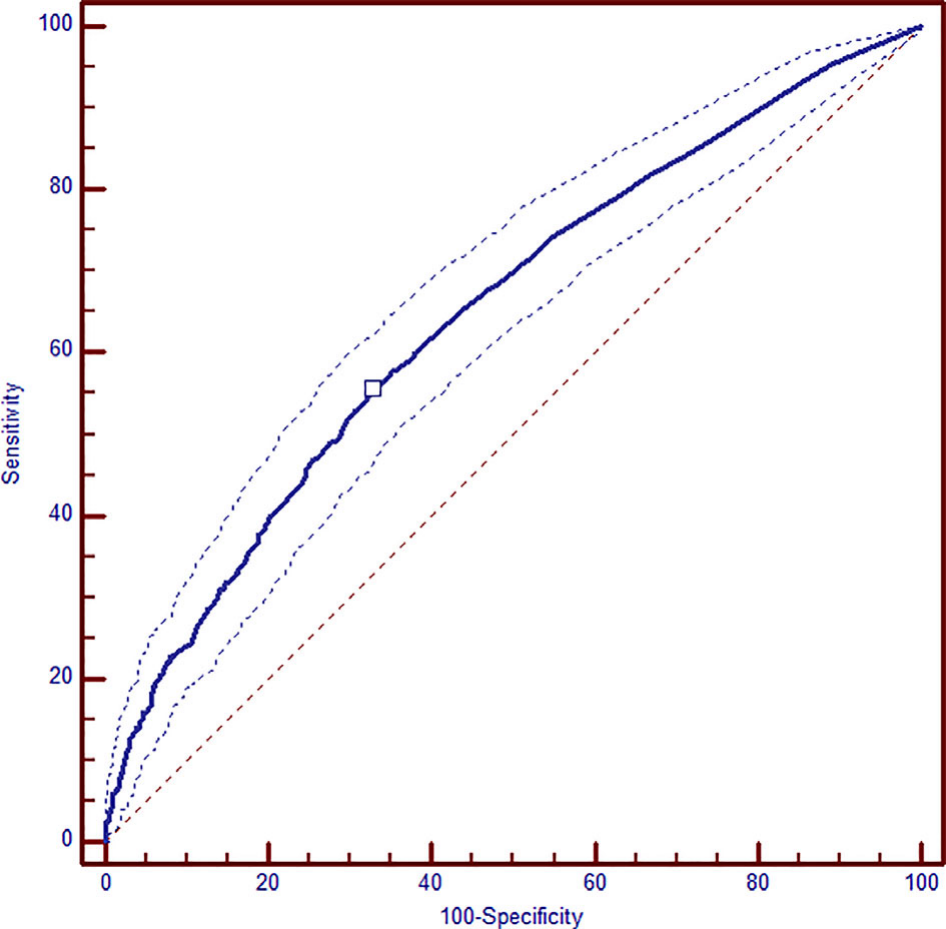
ROC analysis of combined maternal and metabolic significant variables: altered FPG at OGTT, pre-gestational BMI >30, age >35 years, familiar history of diabetes, ART pregnancy, previous GDM, hypothyroidism. AUC 0.647 (95% CI 0.626–0.668); sensitivity 55.3% and specificity 67.3%.

After multivariate logistic regression analysis, pre-pregnancy BMI >30 (OR 1.86, 95% CI 1.42–2.45), family history of diabetes (OR 1.14, 95% CI 1.01–1.29), previous GDM (OR 1.81, 95% CI 1.36–2.43), hypothyroidism (OR 1.52, 95% CI 1.07–2.15), and ART pregnancy (OR 1.81, 95% CI 1.19–2.73) were identified as maternal variables significantly associated with the need of insulin therapy. Similarly, patients who needed insulin therapy were significantly older than women in nutritional therapy (age >35 y OR 1.23, 95% CI 1.02–1.48) and they had more frequently altered FPG at OGTT (OR 1.62, 95% CI 1.34–1.96) (**Table 3**). By integrating all the maternal variables found to be significantly associated to the need of insulin therapy, a ROC curve analysis was performed and the prediction model based on these seven maternal risk factors (altered FPG at OGTT, pre-gestational BMI>30, age >35 years, familiar history of diabetes, ART pregnancy, previous GDM, hypothyroidism) showed acceptable screening performance. The area under curve (AUC) was 0.647 (95% CI 0.626–0.668) and the model has 55.3% sensitivity and 67.3% specificity (**Figure 1**).

**Table 3:** Variables significantly associated to insulin treatment for GDM from multivariate stepwise logistic regression.

Variable	Odds Ratio	95% CI
Altered FPG at OGTT	1.6176	1.3371–1.9568
Pre pregnancy BMI >30	1.8630	1.4159–2.4513
Age >35	1.2259	1.0176–1.4768
Family history DM	1.1433	1.0138–1.2894
ART pregnancy	1.8060	1.1940–2.7318
Previous GDM	1.8141	1.3558–2.4272
Hypothyroidism	1.5197	1.0728–2.1527

FPG, fasting plasma glucose; OGTT, oral glucose tolerance test; BMI, body mass index; DM, diabetes mellitus; ART, assisted reproductive technologies; GDM, gestational diabetes mellitus.

Focusing on the 1,012 patients treated with both diet and insulin, we identified 626 (61.8%) women in therapy with long-acting analogues (Detemir), 286 (28.3%) who received both long and rapid analogues, and 100 (9.9%) patients on rapid analogue only treatment.

Maternal characteristics of the three groups are reported in **Table 4**. Patient who needed combined therapy and rapid insulin analogue were older than those who received only Detemir (D 34.2 ± 5.4 *vs* D+R 35.4 ± 5.5 *vs* 35.3 ± 4.8 years; p = 0.005). Furthermore, patients treated with Detemir or combined therapy had higher pre-pregnancy weight (D 68.0 ± 16.3 *vs* D+R 71.1 ± 17.3 *vs* R 61.5 ± 9.9 kg; p < 0.001) and were more likely to be obese (D 17.9% *vs* D+R 20.9% *vs* R 4%; p = 0.001). They also had more frequently history of previous GDM (D 44.5 *vs* D+R 47.8 *vs* R 10%; p = 0.008). We didn’t find any difference between the three groups in terms of family history of diabetes, pluriparity, chronic hypertension, ART pregnancy, and hypothyroidism.

**Table 4:** Maternal characteristics, including anthropometric data, family and clinical history, of women with GDM treated with long-acting analogues (Detemir) or rapid analogues or combined therapy.

Maternal characteristics	Detemirn = 626	Combined therapy n = 286	Rapid Insulin analoguen = 100	p value
Age (y)	34.2 ± 5.4	35.4 ± 5.5	35.3 ± 4.8	0.005
Pre-pregnancy weight	68.0 ± 16.3	71.1 ± 17.3	61.5 ± 9.9	<0.001
BMI >30	112 (17.9%)	60 (20.9%)	4 (4.0%)	0.001
Family history DM	183 (29.2%)	99 (34.6%)	27 (27%)	ns
Pluriparity	264 (44.5%)	130 (47.8%)	40 (40.0%)	ns
Previous GDM	264 (44.5%)	130 (47.8%)	10 (10.0%)	0.008
ART pregnancy	41 (6.5%)	26 (9.1%)	7 (7.0%)	ns
Chronic Hypertension	16 (2.6%)	–	–	
Hypothyroidism	57 (9.1%)	31 (10.8%)	12 (12.0%)	ns

Values are expressed as mean ± SD or n(%). BMI, body mass index; DM, diabetes mellitus; ART, assisted reproductive technologies; GDM, gestational diabetes mellitus.

Regarding OGTT values, women who required combined long-acting and rapid analogues therapy had higher basal and post load values than those treated with only rapid analogues (basal value: D+R 92.05 ± 27.38 mg/dl *vs* R 83.65 ± 8.65 mg/dl; p = 0.0023. 1 h value: D+R 184.67 ± 49.25 mg/dl *vs* R 180.67 ± 32.97 mg/dl; p < 0.0001. 2 h value: D+R 149.10 ± 48.61 mg/dl *vs* R 145.92 ± 30.83 mg/dl; p < 0.0001). Basal values at OGTT were higher in Detemir group compared with rapid insulin analogue alone (D 92.08 ± 21.44 mg/dl *vs* R 83.65 ± 8.65 mg/dl; p = 0.0011), 2 h post load glucose values were instead higher in short- *vs* long-acting analogues groups (D 133.78 ± 47.27 mg/dl *vs* R 145.92 ± 30.08 mg/dl; p = 0.021) (**Table 5**).

**Table 5:** OGTT values among women with GDM according to different insulin treatment strategy.

	Detemir	Combined therapy	Rapid insulin analogue	p value
Basal	92.08 ± 21.44	92.05 ± 27.38	83.65 ± 8.65	D+R *vs* R = 0.0023 D *vs* R = 0.0011
1 h	170.51 ± 48.02	184.67 ± 49.25	180.67 ± 32.97	D+R *vs* D = <0.0001 D *vs* R = 0.058
2 h	133.78 ± 47.27	149.10 ± 48.61	145.92 ± 30.83	D+R *vs* D = <0.0001 D *vs* R = 0.021

Adjusting for confounding factors, pre-pregnancy BMI < 25 (OR 2.77, 95% CI 1.69–4.55), and normal fasting plasma glucose values at OGTT (OR 2.77, 95% CI 1.68–4.26) were found to be two statistically significant variables for the use of rapid insulin analogue alone (**Table 6**).

**Table 6:** Variables significantly associated to rapid analogues insulin treatment for GDM from multivariate stepwise logistic regression.

Variable	Odds Ratio	95% CI
Normal FPG at OGTT	2.67	1.68–4.26
Pre-pregnancy BMI <25	2.77	1.69–4.55

FPG, fasting plasma glucose; OGTT, oral glucose tolerance test; BMI, body mass index.

The multivariate logistic regression analysis identified altered 1-h plasma glucose level at OGTT (OR 1.51 95% CI 1.11–2.04), 2-h glucose level (OR 1.39 95% CI 1.03–1.88), age >35 years (OR 1.61 95% CI 1.19–2.17), and previous GDM (OR 1.62 95% CI 1.10–2.39) as independent predicting factors for the use of combined therapy with rapid and long-acting analogues for glycemic control during pregnancy (**Table 7**).

**Table 7:** Variables significantly associated to the combined long-acting and rapid analogues insulin treatment for GDM from multivariate stepwise logistic regression.

Variable	Odds Ratio	95% CI
Altered 1° h value	1.51	1.11–2.04
Altered 2° h value	1.39	1.03–1.88
Age >35 years	1.61	1.19–2.17
Previous GDM	1.62	1.10–2.39

Finally, we compared numbers of injections in the three groups and we observed an average of 1.81 (± 0.39) in Detemir group, 4.07 (± 1.00) in combined therapy group and 2.46 (± 0.80) in rapid analogue group. The number of shots was significantly lower in Detemir group compared with combined therapy group (p < 0.0001, estimate difference −2.27), in Detemir group compared with rapid group (p < 0.0001, ED −0.65) and in rapid group compared with combined therapy group (p < 0.0001 ED −1.62).

The analysis of perinatal outcomes showed no differences in terms of gestational age at delivery, preterm birth, mode of delivery, SGA and LGA rate, birthweight >4,000 g, and Apgar score at 10 min between women on NT *versus* those on NT and insulin, and among groups according to type of insulin treatment. However, a higher induction of labor rate was found (NT 29.7% *vs* NT+insulin 41% p < 0.001) in women treated with insulin, especially if under combined long-acting and rapid insulin analogues (D 24.6% *vs* D+R 33.6% *vs* R 27%, p = 0.009) (**Supplementary Tables 1** and **2**).

## Discussion

The present study shows relevant differences between GDM women according to the treatment they need, highlighting that, since the diagnosis of GDM, it is possible to identify those who would be more likely to be treated by insulin and which type of regimen would fit better for them. The identification of specific maternal and OGTT features at diagnosis of GDM would allow to develop orientated therapeutic strategies, in order to timely personalize the nutritional and medical approach to GDM.

The significant variables associated to insulin therapy were maternal age, obesity, previous GDM, altered FPG at OGTT, hypothyroidism, ART pregnancy, family history of DM. Our findings were consistent with previous studies (12, 14, 17) and this risk factors profile identifies a phenotype which is already available at the diagnosis of GDM, allowing to adequately counsel the patient on the increased risk of need for insulin treatment to control glucose, if NT would not be sufficient.

In a retrospective cohort study Meshel reported similar risk factors for insulin therapy (GMD in previous pregnancy, pre-pregnancy obesity, and maternal age >30 y). Only fasting glucose values >95 mg/dl was found to predict insulin therapy but no other abnormal values at OGTT (17). Although not associated with significant differences in maternal and neonatal outcomes, a poor metabolic profile demonstrated by fasting hyperglycemia at OGTT predicted a greater need of insulin therapy and a higher risk of impaired glucose tolerance persistence after childbirth (18). Obesity is a well-known risk factor for GDM and insulin treatment, due to increased insulin resistance (19, 20). Also hypothyroidism has been recently demonstrated to be associated with increased risk of GDM, as thyroid hormones affect glucose regulation during pregnancy and their abnormality may contribute to insulin resistance, aside from the specific role played by thyroid autoimmunity (21). Furthermore, as shown by our results, ART seems to be an independent prognostic factor predicting insulin therapy in women affected by GDM (22–24).

Barnes indicated a model to predict the need of insulin therapy based on maternal characteristic and metabolic profile demonstrating that the number of risk factors was associated with worse pregnancy outcomes (12) and the prediction model had an acceptable accuracy (AUC 0.71 (95% CI 0.693–0.731), even though this does not represent the optimal prediction. We obtained similar results with our prediction model, integrating all the maternal and metabolic significant variables associated to the need of insulin. Nonetheless, Ducarme failed to combine maternal characteristics and biological parameters to develop an accurate model (13).

Regarding the importance of metabolic profile at OGTT, alongside maternal variables, another retrospective analysis in which 1 h and 2 h glucose values at OGTT and the number of abnormal values in 75 g OGTT predicted the requirement of insulin therapies, confirming our findings (14). In the present study population, not only an altered FPG was significantly associated with the failure of NT, but also 1-h glycemic value at OGTT and the risk was higher if one, two, or three values were altered. In those cases, a combined long-acting and rapid analogues therapy was necessary to keep under control glycemic values. On the contrary, the phenotype of a GDM women with normal BMI and normal FPG is significantly associated with the need of only rapid insulin analogues, if NT has failed.

Focusing on insulin therapy we achieved optimal glycemic control with Detemir in 60% of GDM women reducing numbers of daily injections, hypothesizing a better maternal compliance and adherence to therapy. A recent meta-analysis showed no differences between Detemir and Neutral Protamine Hagedorn in term of glycemic control and fetal outcomes in GDM women (5). The use of Detemir in pregnancy is considered safe because it doesn’t cross the human placenta and, unlike Galrgine, it has low affinity for the IGF-1 receptor and this could prove beneficial in the treatment of pregnant women, particularly as regards the effect on birthweight and maternal complications (25).

Data on the use of Detemir in women with GDM are increasing, showing that it allows to achieve a good glycemic control, similar to NPH (26, 27). Studies about Detemir and type 1 diabetes in pregnancy published in recent years showed that Detemir is as effective as NPH when used as a basal insulin in pregnant women affected by type 1 diabetes with the potential to offer some clinical benefits in terms of fasting plasma glucose control (28–30). Detemir is as well tolerated as NPH regarding perinatal outcomes and no safety issues were identified. Results from a recent large international prospective cohort study will provide updated information on the occurrence of malformations and perinatal outcome in women under Detemir treatment compared to other long-acting insulins (31).

By using long-acting analogue treatment fasting glucose values may be normalized and this goal seems to be the major determinant of pregnancy and neonatal outcomes. Furthermore, the use of one injection every 12 h allows to provide a stable level of insulin all-day, managing to drop also postprandial glycemic values, with an irrelevant risk of hypoglycemia. In this scenario postprandial glucose values may play a key role in determining fetal overgrowth in women with severe GDM that require combined therapy (8).

According to our results, we found that women who required long-acting analogues or combined therapy presented a specific phenotype: they more often older, affected by GDM in previous pregnancy, and more insulin resistant (elevated fasting glucose values and more altered OGTT) compared to women who required only rapid analogues who were slimmer and had normal fasting glycemic values. Similar findings are reported in a recent multicenter prospective cohort study (32), where GDM subtypes with different degrees of insulin resistance had distinct phenotype and risks of adverse pregnancy outcomes. Women with GDM and normal insulin sensibility could be assimilated to normal glucose tolerance women in terms of adverse outcomes and pre-pregnancy characteristics. Moreover, women affected by GDM with higher insulin resistance represented a more adverse metabolic profile with greater risk of adverse pregnancy outcomes.

Risk factors for insulin therapy identified in our population are concordant in recognizing a poor metabolic profile that can be the substrate of maladaptation to the insulin resistance state physiologically induced by pregnancy. That is especially true for patients who required combined therapy, that was at high risk of poor glycemic control according history and metabolic factors.

Therefore, it is important to promptly identify patients who need insulin therapy in order to start adequate treatment as soon as possible improving glycemic control and avoiding adverse pregnancy outcomes. Women with insulin resistance and metabolic syndrome appears to be the highest risk group, that requires a strict management in order to improve fetal and neonatal outcomes.

The strength of this study is that it included a large number of patients in each group and that it was performed in a single center where the screening of GDM and the glycemic goals for treatment have been constant during the study period. The major limitation is the retrospective nature of the study.

In conclusion, some maternal constitutional and metabolic factors of GDM can promptly predict the need for insulin therapy. This approach can identify a high-risk group in GDM patients and can allow the prescription of a timely and appropriate therapy in order to improve obstetric outcomes and reducing the time of fetal exposure to hyperglycemia. This can also improve fetal programming thereby reducing short- and long-term consequences.

## Data Availability Statement

The raw data supporting the conclusions of this article will be made available by the authors, without undue reservation.

## Ethics Statement

Ethical review and approval were not required for the study on human participants in accordance with the local legislation and institutional requirements. The patients/participants provided their written informed consent to participate in this study. The research protocol involved only existing records, based on information routinely collected and stored in a de-identified dataset and hence was considered exempt from ethical review board approval.

## Author Contributions

FM contributed to the conception and design of the study. FL, SO, CS, MR, and SS contributed to patient recruitment and collection of data. SV and FL collaborated to data analysis and first draft writing. FM and FP substantially contributed to interpretation of data. FM, SV, and FP revised the manuscript for important intellectual content. All authors contributed to the article and approved the submitted version.

## Conflict of Interest

The authors declare that the research was conducted in the absence of any commercial or financial relationships that could be construed as a potential conflict of interest.
