# Supplementary material for: Different Gestational Diabetes Phenotypes: Which Insulin Regimen Fits Better?
Source: Front Endocrinol (Lausanne). 2021 Mar 9;12:630903. doi: 10.3389/fendo.2021.630903 (PMC7985539; doi:10.3389/fendo.2021.630903)
Supplement: Supplementary file 1 [file Table_1.docx]

| **Perinatal outcomes** | **Diet**  n=962 | **Diet + Insulin**  n=1012 | p value |
| --- | --- | --- | --- |
| GA at delivery | 38.5 ± 2.1 | 38.6 ± 1.5 | ns |
| Induction of labor | 205 (29.7%) | 277 (41%) | **0.001** |
| Elective CS | 165 (17.1%) | 201 (19.9%) | ns |
| In labor CS | 72 (7.5%) | 76 (7.5%) | ns |
| Emergency CS | 94 (9.7%) | 124 (12.2%) | ns |
| Operative delivery | 46 (4.8%) | 50 (4.9%) | ns |
| Preterm birth < 34w | 23 (2.7%) | 22 (2.5%) | ns |
| SGA (<10° centile) | 126 (13.1%) | 112 (11.1%) | ns |
| LGA (>90° centile) | 103 (10.7%) | 122 (12.1%) | ns |
| Birthweight >4000g | 47 (4.9%) | 47 (4.6%) | ns |
| Apgar score < 7 at 10 min | 27 (3.2%) | 16 (1.8%) | ns |

Supplementary Table 1. Perinatal outcomes of women with GDM treated with only diet and those requiring also insulin.

Supplementary Table 2. Perinatal outcomes of women with GDM treated with long-acting analogues (Determir) or rapid analogues or combined therapy.

| **Perinatal outcomes** | **Detemir**  n=626 | **Combined therapy** n=286 | **Rapid Insuline analogue**  n=100 | p value |
| --- | --- | --- | --- | --- |
| GA at delivery | 38.6 ± 1.5 | 38.5 ± 1.6 | 38.8 ± 1.5 | ns |
| Induction of labor | 154 (24.6%) | 96 (33.6%) | 27 (27%) | **0.009** |
| Emergency CS | 75 (12%) | 37 (12.9%) | 12 (12%) | ns |
| Operative delivery | 30 (4.8%) | 15 (5.2%) | 5 (5%) | ns |
| Preterm birth < 34w | 15 (2.4%) | 7 (2.5%) | 1 (1%) | ns |
| SGA (<10° centile) | 67 (10.7%) | 26 (9%) | 19 (19%) | **0.022** |
| LGA (>90° centile) | 78 (12.5%) | 38 (13.3%) | 6 (6%) | ns |
| Birthweight >4000g | 31 (4.9%) | 13 (4.5%) | 3 (3%) | ns |
| Apgar score < 7 at 10 min | 5 (0.8%) | 2 (0.7%) | 1 (1%) | ns |

*Values are expressed as mean±SD or n(%). CS: cesarean section; GA: gestational age; SGA: small for gestational age; LGA: large for gestational age.*
